# Gastric adenocarcinoma with thyroid metastasis: A case study and literature review

**DOI:** 10.3892/ol.2013.1206

**Published:** 2013-02-22

**Authors:** XINGLAI FENG, LIMING SHENG

**Affiliations:** Department of Radiation Oncology, Zhejiang Cancer Hospital, Hangzhou 310022, P.R. China

**Keywords:** gastric cancer, adenocarcinoma, thyroid neoplasm, immunohistochemistry

## Abstract

Previously, cases of metastatic thyroid cancer were only identified following mortality, by autopsy studies. Incidence of this disease is currently estimated to be between 0.5% in all malignant tumors and 24% in all patients based on autopsy studies. Metastatic thyroid cancer is associated with poor prognosis. In the present study, a 58-year-old male presented with a cervical mass. Subtotal gastrectomy and D2 lymph node dissection identified a poorly differentiated gastric adenocarcinoma. Following this, fine needle aspiration was performed, revealing that the thyroid tumor cells were similar to gastric tumor cells, indicative of metastasis from this organ of origin. In addition, immunohistochemical analysis was consistent with this diagnosis. Palliative radiotherapy of the thyroid mass was performed. At manuscript submission, the patient remained alive.

## Introduction

Distant metastases in liver, lung, bone and brain are commonly identified in gastric cancer patients (1–3). Detection of metastases in the thyroid is uncommon despite the rich vasculature of the thyroid. A previous study on autopsy observations estimated that the incidence of metastatic thyroid cancer ranged between 0.5% in unselected and 24% in widespread malignant patients (4). The most common primary sites of metastases are the kidney, lung, breast and colon (5,6). Metastatic thyroid tumors are often accompanied by synchronous lesions in additional organs and correlate with a poorer prognosis than metastatic thyroid tumors associated with no additional lesions. A number of thyroid metastases are not diagnosed during the lifetime of the patient and fine-needle aspiration (FNA) biopsy has been hypothesized to be suitable for the detection of this disease (7).

A method to distinguish between a metastatic thyroid tumor from a secondary cancer, particularly following a long-term total gastrectomy, is an important aim of research in this field. In the current study, we present a case of metastatic thyroid tumor originating from gastric adenocarcinoma. In addition, a literature review of current studies on this extremely rare metastatic event was performed.

## Case study

A 58-year-old male presented with abdominal pain and melena which was diagnosed as gastric adenocarcinoma in February 2005. The patient underwent subtotal gastrectomy and D2 lymph node dissection, which confirmed a diagnosis of poorly differentiated adenocarcinoma measuring 5x4x4 cm in size. The tumor had invaded the subserosal layer (T3) without lymph node metastases and was classified as T3N0M0, stage III according to the TNM system. Following surgery, the patient received 6 cycles of adjuvant chemotherapy (5-FU and cisplatin) in 3-week intervals with regular follow-up examinations, which included physical examinations, blood counts, liver function tests and abdominal ultrasound.

**Figure 1:**
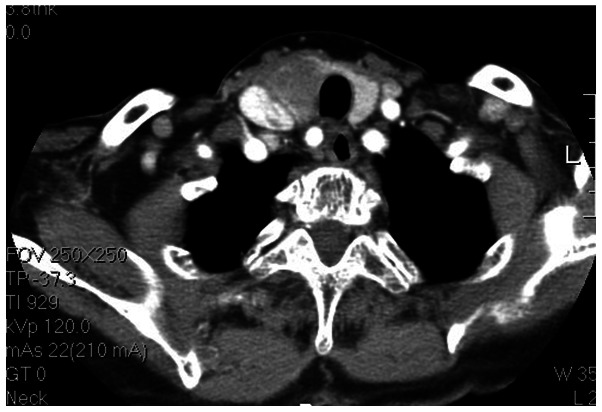
Cervical computed tomography revealed a 3×3×6-cm solid mass in the right thyroid.

Five years later, the patient was hospitalized following observation of a rapidly growing mass in the neck. No other symptoms, including abdominal pain, respiration difficulties or neck pain, were identified. Physical examination identified an enlarged right thyroid lobe and a moderately hard nodule. Thyroid function tests were within normal range and plasma concentration levels of carcinoembryonic antigen (CEA), carbohydrate antigen 125 and α fetoprotein were normal. Thyroid ultrasonography and computed tomography revealed a 3×3×6-cm solid mass in the right thyroid (Fig. 1). No other metastases were identified by chest radiography, brain magnetic resonance imaging and bone scintigraphy. FNA biopsy of the thyroid mass was performed to confirm the histological observations and revealed diffuse infiltration of poorly differentiated tumor cells in thyroid follicles. The morphology of these tumor cells was similar to gastric lesions. Immunohistochemistry of the tumor cells revealed positive staining for CEA and negative for thyroglobulin and thyroid transcription factor-1 (TTF-1). These features are consistent with those of metastatic gastric adenocarcinoma and do not indicate primary thyroid cancer. The patient was treated with palliative thyroid radiotherapy but refused any form of adjuvant chemotherapy.

## Discussion

Thyroid metastases originating from gastric adenocarcinomas are extremely rare in comparison to other gastrointestinal tumors (4). In clinical practice, common primary cancer sites include the kidney, breast and lung (5,6). In the current case study we present a patient with metastatic thyroid cancer manifested as a thyroid mass originating from a primary gastric adenocarcinoma.

To the best of our knowledge, only four cases of thyroid metastases originating from gastric adenocarcinomas have been previously reported in English-language literature (7–10) (Table I). The age of these cases ranged between 39 and 71 years with a median age of 58 years. The male:female ratio was 1:3. The present case was a 58-year-old male patient with primary gastric adenocarcinoma classified as T3N0M0. Absence of lymphomagenesis indicated that hematogenic routes may be associated with thyroid tumor metastases originating from gastric adenocarcinomas. Of the four cases, synchronic and metachronic metastases occurred in two cases. In synchronic metastases patients, the interval between initial primary tumor diagnosis and metastases was variable (range, 4–15 months). In the current study, the latency period of 35 months was the longest of all previously reported cases. Primary cancers were analyzed by hispathological analysis and the majority were identified as poorly differentiated gastric adenocarcinomas, which spread rapidly from the primary site. In 3/4 cases, thyroid function tests were of normal range, and T3 and TSH levels were at the lower end of the normal range in one patient only.

At present, metastatic thyroid tumors are difficult to identify and diagnose as it is hard to determine whether the tumor is primary or secondary, particularly following long-term complete remission. Cytological examination of thyroid specimens obtained by FNA biopsy is an important process in this diagnosis. Analysis of the morphology of tumor cells from the thyroid is the first diagnostic step, followed by immunohistochemical analysis of various markers to determine whether the origin of the cells is the primary site. In the present case study, immunohistochemistry revealed that tumor cells exhibited positive staining for CEA and were negative for thyroglobulin and TTF-1. These observations are consistent with previous studies.

Metastatic thyroid tumor from gastric adenocarcinoma is generally considered a systemic disease with an extremely poor prognosis. In previous clinical cases, the survival of two patients who refused treatment was only one month (7,10). No current management protocol for this diease has been firmly determined by consensus. At present, bilateral total thyroidectomy or chemotherapy have been identified to prolong survival (8,9). In the present study, the patient received palliative radiotherapy and survival was similar to patients who had recieved surgery or chemotherapy. However, the results remain unsatisfied. Patients treated with surgery, radiotherapy and adjuvant chemotherapy might be achieved a better prognosis.

The current study describes a case of thyroid metastatic gastric cancer. Although it is generally accepted that thyroid metastasis of gastric cancer is extremely rare and the prognosis is usually poor, research and communications on this disease must be performed to enhance clinical understanding and patient survival. Immunohistochemical diagnosis is essential for conclusive diagnosis of thyroid metastatic gastric cancer.

## Figures and Tables

**Table I:** Reported cases of metastatic thyroid tumor from gastric adenocarcinoma identified in a review of English-language literature.

			Primary cancer			Metastatic cancer		
Case no. (ref)	Gender	Age (years)	Pathology	Treatment	Interval (months)	Pathology	Treatment	Outcome (months)
1 (7)	F	39	Ade	None	0	Ade	None	1
2 (8)	M	71	Poorly	None	0	Poorly	Bilateral total thyroidectomy	4
3 (9)	F	63	Poorly	Distal subtotal gastrectomy	15	Signet-ring	Chemotherapy	6
4 (10)	F	60	Poorly	Subtotal gastrectomy	4	Poorly	None	1
Present case	M	58	Poorly	Subtotal gastrectomy	35	Poorly	Radiotherapy	5^a^

a Alive. M, male; F, female; Ade, adenocarcinoma; poorly, poorly differentiated adenocarcinoma; signet-ring, signet-ring carcinoma.
